# Liver collision lesion: inflammatory hepatocellular adenoma within focal nodular hyperplasia

**DOI:** 10.1093/gastro/goaa074

**Published:** 2020-12-14

**Authors:** Matteo Renzulli, Irene Pettinari, Francesco Vasuri, Matteo Ravaioli, Beniamino Corcioni, Daniele Spinelli, Giovanni Marasco, Matteo Cescon, Antonietta D’Errico, Rita Golfieri

**Affiliations:** 1 Department of Radiology, IRCCS Azienda Ospedaliero-Universitaria di Bologna, Via Albertoni 15, Bologna, Italia; 2 Pathology Unit, S. Orsola Malpighi University Hospital, Bologna, Italy; 3 General Surgery and Transplantation Unit, Department of Medical and Surgical Sciences, S. Orsola Hospital, University of Bologna, Bologna, Italy; 4 Department of Medical and Surgical Sciences, S. Orsola Hospital, University of Bologna, Bologna, Italy

## Introduction

Focal nodular hyperplasia (FNH) and hepatocellular adenoma (HCA) are both benign nodular hepatocellular lesions. FNH is the second-most common solid liver tumour, representing ∼8% of all primary hepatic neoplasms [1]. If the diagnosis of FNH is firmly established on the basis of imaging, and if the individual is asymptomatic, follow-up imaging is not required, and the patient can be discharged. However, there is a poor association between FNH and symptoms; therefore, even in symptomatic cases, treatment is rarely indicated [2]. HCA is ∼10 times less common than FNH. In contrast to FNH, HCAs have been considered to be a possible indication for surgical resection due to the potential for bleeding and malignant transformation [3].

There have been reports of the simultaneous occurrence of FNH and HCA in the same liver, suggesting a link between these two lesions [4, 5]. However, the association between them in a unique liver mass has rarely been described [6]. Here, we report the case of a liver collision lesion comprising a focal HCA within an FNH.

## Case report

A 23-year-old nulliparous woman with a 3-year history of oral-contraceptive use underwent ultrasound (US) for abdominal discomfort in January 2017. A 5-cm focal liver lesion was found in the left lobe, showing features of FNH. Contrast-enhanced US was not performed because its highest diagnostic accuracy is only demonstrated in FNHs of <3 cm according to the European Association for the Study of the Liver (EASL) Clinical Practice Guidelines on the management of benign liver tumours [2]. Therefore, magnetic resonance imaging (MRI) was conducted, as it may achieve a correct diagnosis based on its high diagnostic performance [2].

**Figure 1.**
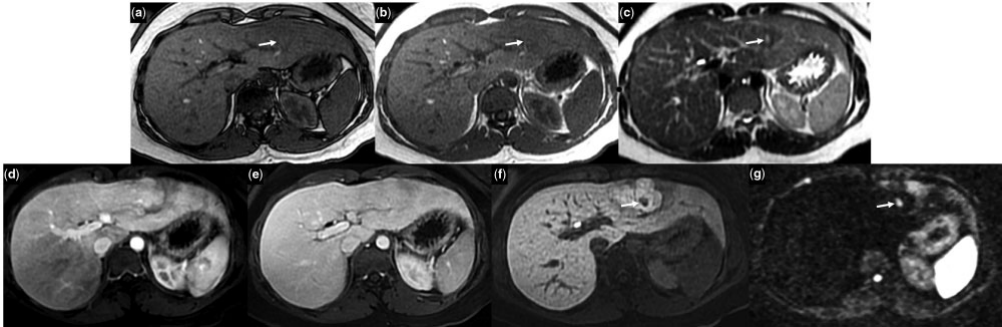
Magnetic resonance imaging demonstrating a nodule measuring 1.1 cm with smooth margins within a 6-cm mass with typical imaging features of focal nodular hyperplasia. This nodule presented the absence of signal dropout on opposed-phase T1-weighted image (arrow in a) in contrast to the in-phase T1-weighted image (arrow in b), strong hyperintensity on T2-weighted image differently from the adjacent lesion (arrow in c), hyperintensity in the arterial phase equal to the adjacent lesion (d), and no washout of contrast media in the venous phase in which it appeared isointense to the adjacent lesion (e). The nodule demonstrated strong hypointensity in the hepatobiliary phase (arrow in f) and strong restriction on diffusion-weighted image (arrow in g) compared with the adjacent lesion. These magnetic resonance findings were strongly suggestive for inflammatory hepatocellular adenoma.

MRI with gadoxetic acid, a hepatospecific contrast agent, revealed a 6-cm liver mass in the left lobe with almost all imaging features typical for FNH according to the EASL guidelines [2] (**Figure 1**). However, MRI demonstrated the absence of lesion homogeneity—a unique imaging characteristic not typical for FNH. In fact, within the mass, a 1.1-cm nodule with smooth margins was detected. This small nodule showed different imaging characteristics from those of the lesion in which it was embedded (**Figure 1**). According to the EASL guidelines [2], the likely imaging diagnosis of this smaller nodule was inflammatory HCA (I-HCA). Therefore, the final supposed diagnosis of the entire mass based on imaging results was a liver collision lesion: I-HCA surrounded by FNH.

Laboratory examination identified no anomalies. In particular, liver-function test results and serum α-fetoprotein and carcinoembriogenic antigen levels were normal. After unanimous consensus by a benign-liver-tumour multidisciplinary team, the patient underwent left hepatectomy. This treatment was chosen for the following reasons: (i) the diagnosis of FNH was based on a combination of typical imaging features according to the EASL guidelines [2], which were not present in our case; (ii) biopsy of the atypical nodule within the mass was not possible owing to its small dimensions (approximately 1 cm) and difficulties in detecting this small target lesion on US examination; and (iii) the patient was symptomatic.

Gross examination of the surgical specimen after left lobectomy revealed a 6-cm lesion, with sharp margins, which was yellowish and had a central scar. On histology, the lesion mostly comprised benign-looking hepatocytes forming nodules surrounded by fibrotic septae, with bile duct reaction and dystrophic/teleangectatic vessels, all of which are typical features of FNH. Within the mass, a small nodule approximately 1 cm in diameter was confirmed. The nodule was histologically characterized by the proliferation of benign hepatocytes arranged in a trabecular pattern, with focal atrophy of hepatocytes and without significant fibrosis but with teleangectatic vessels, more similar to an HCA. Immunohistochemistry for glutamine synthetase showed a strong ‘map-like’ positivity in the main lesion; however, expression was lost in the minor area, confirming the final diagnosis of FNH associated with focal I-HCA.

The post-operative course was uneventful, and the patient was discharged in a good condition on the fifth day after operation. Ten months after the surgical intervention, the patient remained asymptomatic with normal hepatic-function test results. Follow-up MRI showed liver regeneration without signs of tumour relapse.

## Discussion

One case of a collision liver tumour similar to ours was previously published; however, that case comprised HNF1A-inactivated HCA and classic FNH [6]. In addition, the previous case did not report imaging features. It is well known that ever-increasing knowledge in the field of HCA classification is not accompanied by similar advances in the field of imaging diagnosis of HCA; therefore, the diagnosis of HCA other than the steatotic form becomes more complex [7]. Certainly, MRI performed with hepatospecific contrast media represents the technique of choice for many focal liver lesions, facilitating the identification of the lesion phenotype [7, 8]. However, in the present case, an adenomatous lesion was embedded in a different larger lesion, making the overall imaging diagnosis more complex. This report underlines the importance of evaluating a lesion in its entirety, as new imaging techniques allow the assessment of many features, facilitating the identification of liver collision lesions.

## Authors’ contributions

M.Re., I.P., and R.G. conceived and designed the project. B.C., D.S., M.Ra., M.C., A.D., and G.M collected the data. All authors drafted the manuscript. All authors read and approved the final manuscript.

## Funding

No financial supports to declare.
